# Care challenges of home health patients living with dementia: a pathway forward with palliative care

**DOI:** 10.1186/s12904-023-01247-9

**Published:** 2023-08-29

**Authors:** Connie S. Cole, Ashley Dafoe, Caroline K. Tietbohl, Sarah R. Jordan, Amy G. Huebschmann, Hillary D. Lum, Christine D. Jones

**Affiliations:** 1 Division of General Internal Medicine, Department of Medicine, University of Colorado Anschutz Medical Campus, Aurora, CO USA; 2 Adult and Child Center for Outcomes Research and Delivery Science, School of Medicine, University of Colorado, Children’s Hospital Colorado, Aurora, CO USA; 3 Department of Family Medicine, University of Colorado School of Medicine, Aurora, CO USA; 4 Division of Healthcare Services, Molina Healthcare of Illinois, Oak Brook, IL USA; 5 Ludeman Family Center for Women’s Health Research, Department of Medicine, University of Colorado School of Medicine, Aurora, CO USA; 6 Division of Geriatric Medicine, Department of Medicine, University of Colorado Anschutz Medical Campus, Aurora, CO USA; 7 Denver-Seattle Center of Innovation for Veteran-Centered and Value Driven Care, Veterans Health Administration, Eastern Colorado Health Care system, Aurora, CO USA; 8 Division of Hospital Medicine, Department of Medicine, University of Colorado, Aurora, CO USA

**Keywords:** Palliative care, Home health care, Dementia, Alzheimer’s disease, Qualitative methods

## Abstract

**Background:**

Home health care (HHC) is a leading source of care support for older adults with serious illness, particularly patients living with dementia (PLWD). Demand for HHC is expected to continue to grow, driven by an aging population and preference for non-institutional care. HHC agencies are frequently under pressure to find effective approaches for improving care delivery and quality. One strategy that has the potential to improve the quality of life and patient satisfaction in HHC for PLWD is the integration of palliative care. Therefore, we sought to understand the experiences and needs of PLWD and their family caregivers specifically focusing on ways that HHC and palliative care may be integrated as part of the care transition from hospital to home, to better support PLWD and their families.

**Methods:**

We conducted a descriptive qualitative study focusing on the perspectives of patients, caregivers, and healthcare team members about palliative care delivery for patients receiving HHC. Interviews were audio-recorded and professionally transcribed. In this analysis, we specifically report on dementia-related content using an iterative, team-based thematic analysis approach.

**Results:**

We identified three themes: 1) ’Living in the Whirlwind’ which describes the many competing demands on caregivers time and the associated feeling of loss of control, 2) ’Thinking Ahead’ which describes the importance of thinking beyond the day-to-day tasks to begin planning for the future, and 3) ‘Pathways Forward’ which describes the integration of palliative care into HHC to provide enhanced support for PLWD and their caregivers.

**Conclusion:**

In this qualitative study, our formative work identified the importance of providing anticipatory guidance (e.g., safety, advance care planning) coupled with emotional and pragmatic care supports (e.g., finding resources, navigating insurance) to sustain caregivers who are struggling with the whirlwind.

**Supplementary Information:**

The online version contains supplementary material available at 10.1186/s12904-023-01247-9.

## Introduction

In 2022, about 1 in 9 Americans, age 65 and older, were living with Alzheimer’s disease and related dementia (ADRD) [1, 2]. The prevalence of ADRD is expected to rise due to increased cardiovascular risk factors associated with obesity in the United States [1, 2]. ADRD places a heavy load on patients, caregivers, the healthcare system, and society due to need for increased supervision, assistance with making decisions and eventual need for around the clock assistance and long-term care [2].

Home health care (HHC), which is often started following a hospitalization, is a leading source of care for frail older adults, particularly patients living with dementia (PLWD) [3]. In 2020, 3.1 million Medicare beneficiaries received HHC and the large majority were older adults with multiple chronic conditions, including ADRD [4]. Demand for HHC is expected to continue to grow, driven by an aging population and preference for non-institutional care. The revised HHC payment model, the patient-driven groupings model, which was introduced in 2020, coupled with increased demand for HHC, places pressure on HHC agencies to find effective approaches for improving care delivery and quality [5].

One strategy that has the potential to improve the quality of life and patient satisfaction in HHC for PLWD is the integration of palliative care [6]. Defined as holistic care for individuals with serious illness, palliative care aims to improve the quality of life of patients and their family caregivers [7]. Studies examining palliative care for nursing home residents with dementia have demonstrated its potential to improve quality of life and patient satisfaction and to reduce risk of acute care use (hospitalization, emergency department visits) [8, 9]. Integration of palliative care in a home setting for PLWD is under studied and home-based palliative care is not widely integrated into existing HHC delivery in the United States. A hospitalization for a PLWD often results in functional decline and may suggest a worsening of their prognosis. Hospitalization could serve as a timely signal to assess or reassess the role of palliative care.

Therefore, we sought to understand the experiences and needs of PLWD and their caregivers, specifically focusing on ways that HHC and palliative care may be integrated as part of the care transition from hospital to home, to better support PLWD and their families.

## Methods

This work is derived from a primary study which gathered perspectives of patients, family caregivers, and clinicians in order to develop and pilot a model for identifying and addressing palliative care needs through HHC. Methods for the primary study have been previously described in detail [10]. Below is a summary of the procedures for this sub-analysis.

### Study design

This descriptive qualitative study is part of a larger study which gathered perspectives of patients, family caregivers, and healthcare team members to develop and pilot a model for identifying and addressing palliative care needs through HHC. Methods for the primary study have been previously described in detail [10]. Briefly, we employed semi-structured interviews to better understand experiences with HHC, care coordination, and understanding benefits and barriers to implementing palliative supports in HHC (e.g., symptom management, advance care planning, caregiver support, care coordination). The Colorado Multiple Institutional Review Board approved this study.

### Participants & recruitment

The study was conducted with patients receiving HHC in the community and their caregivers, as well as primary care providers (PCPs), home health clinicians, and hospitalists. Participants recruited for interviews were referred for HHC and were felt by either a hospitalist or PCP that they could benefit from additional palliative or transitional care at home. Patients were either contacted by phone in their hospital room and/or provided a flyer about the study during a hospitalization or the flyer was sent by mail if the patient was recruited from an outpatient clinic. The patients were then called for verbal consent for an interview. During interviews, the patient was asked to provide information about up to two caregivers involved in their care. For patients who were determined by their hospitalist or PCP to have cognitive impairment that would preclude them from providing informed consent, the legally authorized representative was contacted instead and invited to participate in the study if they were also involved as a caregiver in the patient’s care team. Patients and caregivers were provided with a $25 gift card for participating in interviews. Methods for healthcare team member recruitment are previously described in detail [10]. Translation and interpretation services were available for non-English speaking potential participants. All participants were recruited from the University of Colorado Hospital or the VA Eastern Colorado Health Care System. Informed consent was obtained from all participants.

### Data collection

All interviews were conducted by phone and audio recorded. The interview guide for patients and caregivers (Appendix 1) and healthcare team members (previously described [10]) was developed using the Practical Robust Implementation Sustainability Model (PRISM) [11]. Patient and caregiver questions included experiences with HHC including symptom management and desired in-home supports. PCP, home health clinician, and hospitalist questions included experiences with identifying and addressing palliative care needs across inpatient, primary care, and home health settings. We did not ask caregivers directly about caring for persons living with dementia but did investigate this further if caregivers spontaneously mentioned it. Interviews were professionally transcribed, de-identified, and uploaded to ATLAS.ti (version 9) software for data management.

### Data analysis

We used an iterative, thematic analysis approach. The codes were applied by two team members of the multidisciplinary team (AD and SJ) with a subset (20%) double coded by another team member (CT). Additional coding was completed by a fourth team member (CC) for this sub-analysis focused on the experiences caring for PLWD. All four team members involved in data coding have qualitative experience. Domains were created from related codes and themes were developed based on observed patterns describing the phenomenon of interest.

## Results

We conducted 52 interviews with 53 individuals (one patient/caregiver dyad interview). Healthcare team participants included inpatient providers (n = 11) (physicians, nurse practitioner, social worker), PCPs (n = 17) (physicians, nurse practitioners, registered nurses, social workers), and HHC providers (n = 10) (nurses, social workers, occupational therapists, and leaders) at local home health agencies. Patient (n = 4) and caregiver (n = 10) interviews included 8 caregivers for PLWD. Overall, each group identified challenges facing older adults living with dementia and the increased need for supports to remain at home. Participant characteristics are presented in Table 1 (healthcare team participants) and Table 2 (patients and caregivers).

**Table 1:** Characteristics of Clinician Participants

Characteristic	Mean (range)		N (%)
**Age**	47 (30–74)		
**Gender**			
Male			9 (23.7%)
Female			29 (76.3%)
**Race/Ethnicity**			
White			31 (81.6%)
Asian			6 (15.8%)
Multiracial			1 (2.6%)
**Setting/Discipline**			
Inpatient Physician Nurse Practitioner Social worker			11 (28.9% 9 (81.8%) 1 (9.1%) 1 (9.1%)
Primary care			17 (44.7%)
Physician			11 (64.7%)
Nurse Practitioner			2 (11.8%)
Social worker			3 (17.6%)
Other			1 (5.9%)
Home Health			10 (26.3%)
Leadership (administrators, managers, directors)			4 (40%)
RN/PT/OT			2 (20%)
Social work			2 (20%)
Hospice			2 (20%)
**Facility**			
Academic health system			20 (71.4%)
VA medical center			8 (28.6%)
**Highest Degree**			
Associate			3 (7.9%)
Bachelor’s			0 (0%)
Master’s			11 (28.9%)
Doctoral			23 (60.5%)
Other			1 (2.6%)

**Table 2:** Characteristics of Patient and Caregiver Participants

Characteristic	Mean (range)		N (%)
**Patient Age**	74 (66–81)		
**Patient Gender**			
Male			3 (75.0%)
Female			1 (25.0%)
**Patient Race**			
White			3 (75.0%)
Black			1 (25.0%)
**Caregiver Age**	58 (46–82)		
**Caregiver Gender**			
Female			10 (100%)
**Caregiver Race/Ethnicity**			
White			6 (60%)
Asian			1 (10%)
Black			1 (10%)
Hispanic			1 (10%)
Multiracial			1 (10%)
**Length of Caregiving**			
< 5 years			5 (50%)
5–10 years			2 (20%)
10–20 years			2 (20%)
20 + years			1 (10%)
**Caregiver Relationship to Patient**			
Child			8 (80%)
Spouse			1 (10%)
Friend			1 (10%)

We identified three themes: 1) ’Living in the Whirlwind’ which describes the many competing demands on caregivers time and the associated feeling of loss of control, 2) ’Thinking Ahead’ which describes the importance of thinking beyond the day-to-day tasks to begin planning for the future, and 3) ‘Pathways Forward’ which describes the integration of palliative care into HHC to provide enhanced support for PLWD and their caregivers.

### ‘Living in the Whirlwind’

‘Living in the Whirlwind’ refers to the numerous competing demands that a caregiver must pay attention to every day. It often includes the activities of caring for children and parents, working, paying the bills, managing healthcare visits, and dealing with crises that emerge. For example, family caregivers had to navigate unexpected challenges such as when a paid caregiver gets sick and is not able to work. Caregivers described the whirlwind as all the urgent tasks that need to be completed, but which can keep caregivers from having time and energy for other caregiving priorities that may become urgent in the future – such as preparing for how to handle further progression of dementia. Caregiver statements in this theme reflect the experience of being overwhelmed by the demands of providing care for family members living with dementia. The process was described as “extremely challenging,” “too much,” “not easy,” “overwhelming,” leading caregivers to feel “at wit’s end,” and even contributing to hospitalization of PLWD when caregivers were burned out and no longer able to cope.

One caregiver described being caught between providing care for her mother with dementia, diabetes, and high care needs while simultaneously providing care for a daughter that was recently hospitalized with COVID, all while continuing to work:

*“I just got to…I’m just trying to keep myself healthy…That’s the biggest concern because if I get sick then nobody gets help…And then, my daughter is here now from the hospital, and she seems to be doing pretty good. … She needs physical therapy too… I’m trying to focus on both…I have to be on the phones by 8:30 and ready to go, you know, they’re [work] not as understanding. They don’t know what it’s like taking care of somebody with Alzheimer’s” (Caregiver-148).*

Another participant described caring for both parents who require help with meals and activities of daily living, placing a high burden on family and paid caregivers to prepare meals and keep them safe. She described difficulties finding reliable paid home caregivers, including the lack of coverage if one of the paid caregivers misses work. These difficulties led to her feeling forced to work less, describing:

*“This whole situation often has me at wit’s end. I have had to go part time in my work and, it’s been extremely challenging…I am fortunate in the resources that I have. I don’t know what people do” (Caregiver-141).*

A third participant described her father as rapidly worsening and needing more assistance with ‘memory care.’ She indicated that she was struggling to keep up with her father’s finances and Medicare as well as her own finances, children, and household chores:

*“A huge issue that I am having is being able to keep up with all of his finances and my finances and take care of having a son in the house and then there’s the house to take care of and there is all these things to do and it’s like oh God, I got to figure it out. I just don’t have time” (Caregiver-130).*

Clinicians expressed similar viewpoints and provided insight into the problems that can arise from caregiver burnout. One primary care clinician stated:

*“…the caretaker also needs a lot of support …I think more so to me, sometimes the caretakers need more support than the patient” (PCP-14)*.

This insight was also described by a hospitalist:

*“how we do caregiver relief and support because it is really hard to deal with a loved one who has dementia …we unfortunately have people admitted who have dementia…not because they have any physical, medical need to be in the hospital but it’s because their family is burnt out and they can no longer take care of the person because physically they’re wandering too much, they’re combative…just totally without resources and at their wit’s end… Maybe we could have prevented this hospitalization which is traumatic for everyone …if [we could] address that earlier on, [and] provided resources that might have prevented [the hospitalization]” (Hospitalist-25).*

An illustration of this was provided by one family caregiver which led to a 911 call:

“*it was a fight with her with the bathtub and I kept saying mom don’t keep getting in the bathtub. You need to get you a shower chair and sit in there and take a shower and she didn’t want to listen, so she ended up being stuck in the bathtub. … and we had to call the 911 to get her out of the tub” (Caregiver-148).*

### ‘Thinking ahead’

The need for ‘thinking ahead’ was identified by clinicians in this study and refers to the need for clinicians to help patients and caregivers anticipate events and prepare ahead of time. Clinicians indicated that the slow, unpredictable trajectory of dementia can lead caregivers to experience special challenges and need support to think ahead. Clinicians frequently mentioned advance care planning as an important task to complete soon after diagnosis while patients still have the capacity to participate and make their preferences known. One PCP indicated:

“O*ften we hear, ’Oh, I wish I would’ve known this when’ …so just really more information up front so that when people are making decisions and trying to plan with their families and trying to think about what somebody wants, they have information up front”* (PCP, NP, VA-32).

Identifying safety needs related to dementia and preparing for the eventual need for around the clock care were also mentioned as important areas for future planning.

*“I mean probably they need to start doing long term care planning as soon as possible…care can be very costly especially if it goes on for long periods of time and not all of our seniors are even [able to afford care]…many of them have their resources to support themselves for, you know, ten, fifteen years with caregivers in place. So I think the families really need to start talking about, ‘can we afford this care and what kind of care can we afford?’ The sooner the better, because you don’t want to have to start looking for assisted living options or just other options when it’s too late and then you realize you don’t have the money, or the patients don’t have the money to care for themselves.” (*HHC Administration-1).

While the need for ‘thinking ahead’ was identified primarily by clinicians, one caregiver described an instance when lack of planning led to a potential crisis when safety needs were not identified, and her mother wandered down the basement steps.

“…*we are going today to put child’s knob on the doors because yesterday she ended up opening the door to the basement. Somehow, she made it down there without falling so that was scary*” (Caregiver-148).

Ultimately thinking and preparing ahead of time, such as putting safety devices into place, can avert a potential crisis.

Moreover, sometimes even when planning ahead, caregivers faced difficulties finding and accessing resources. For example, one caregiver found that their respite care needs did not align with what was available:

*“They have to do it [respite in facility] for like 30 days which is a long time. I mean if I need just a 2-week vacation, you can’t do that. You have to pay to get her into a facility and they don’t want her there for just two weeks. They want her there for a month and it’s like, I just need a break for two weeks”* (Caregiver-153).

### ‘Pathway forward’

Both patients, caregivers and clinicians described integration of palliative care supports that would be identified, coordinated and provided within HHC as one ‘pathway forward’ to assist caregivers in managing the whirlwind, thinking ahead, and avoiding burnout. Helpful pragmatic supports identified in this study include respite care, care coordination, anticipatory guidance, and emotional support. Clinicians described palliative care as supporting caregivers with gentle guiding discussions. Anticipatory guidance is important in helping caregivers know what to expect, make needed plans and prepare for future outcomes, rather than react to unexpected crises. One PCP referred to palliative care as:

*“extra eyes and ears in the home that, you know, if you deteriorate this is what they can help you with and help you get extra care in the home, help manage your symptoms and that sort of thing*” (PCP-10).

Advance care planning, a central tenet of palliative care, is not a typical service or support offered by home health care agencies but was discussed as an important service to integrate with HHC for PLWD and their caregivers. Clinicians stressed the need to begin advance care planning to discuss, think, and plan for future care while the patient is still able to have such conversations and make their preferences known. Making their values and preferences known early may help families to advocate for them when they become unable to advocate for themselves. PCPs felt there was a role for HHC to prepare patients and caregivers for advance care planning conversations with their PCP, while some caregivers thought it might be confusing for PLWD to have advance care planning conversations with HHC. For example, one clinician stated:

*”I think palliative care always makes a difference. I think they really can be helpful in so many areas but one of which is …helping the patient to understand the gravity of their illness. And what their goals of care are…in the remaining time that they likely have left; to the ability that we’re able to know that…we’re hopefully wrong a lot of the time and people can live longer but that’s often not the case so I think palliative care getting involved … would have given the family more resources in terms of who to talk to … [when] things seem dire”* (PCP-21).

## Discussion

In this descriptive qualitative study, we found the feeling of ‘Living in the Whirlwind’, being “overwhelmed” and at “wit’s end” was common among caregivers as they balance caring for family members living with dementia with their family and work responsibilities. Our participants felt that increased availability of palliative care supports in the home could help address care challenges for PLWD, and their caregivers.

Consistent with prior research, the participants in our study revealed critical gaps in support for caregivers of PLWD during HHC. Key gaps included unmet palliative care needs such as advance care planning, navigating financial matters with insurance, accessing respite care, and understanding ADRD disease progression [12]. Caregiving tasks include helping with chores and shopping, managing finances, providing transportation, helping with medications, assisting with personal care (e.g., bathing, dressing), and managing behavioral symptoms of the disease (e.g., wandering, nighttime disturbances). Much of this care is provided in the community, often by women who may experience higher levels of burden as “sandwich generation” caregivers for both parents and children, as was described in our study [13]. Participants in our study made it clear that more palliative support is needed for PLWD and their caregivers. Burgdorf et al., recently proposed a new model of assessment and support in HHC which includes proactively asking about caregivers needs and sharing this information within the medical record [12]. Identifying caregivers that are struggling is a start, but more comprehensive caregiver support is needed, particularly as the numbers of individuals living with dementia grows.

Healthcare team members in our study identified the lack of these supports as contributing to hospitalization of the older adult when caregivers are no longer able to cope. PLWD are at greater risk of experiencing a preventable hospitalization including an unplanned hospital readmission within 30 days of discharge [2]. Hospital admissions have physical and emotional costs for patients and their families, result in iatrogenic complications, including cognitive and functional decline, and contribute to the rising costs of healthcare [14, 15]. At least some of these hospitalizations are potentially preventable, and care models incorporating palliative care principles hold promise and have reduced health care utilization and improved quality of care delivery in hospitals, nursing homes, and skilled nursing facilities [8, 16]. However, care models that incorporate palliative care into HHC are needed.

Integrating palliative care into home health can be beneficial for patients with serious illness, but it comes with various challenges. HHC agencies may have limited resources and funding to implement and sustain comprehensive care programs. Palliative care often requires additional staff, specialized training, and supportive services which can strain the financial capacity of HHC agencies. Moreover, HHC staff may require additional training and education to deliver palliative care effectively. Palliative care involves specialized skills in pain and symptom management, communication, and addressing psychological and spiritual needs, which may not be part of standard home health training. Despite these challenges, integrating palliative care into HHC remains essential for improving the quality of life for patients with serious illness, particularly PLWD and their caregivers. Addressing these challenges requires a concerted effort from policymakers, researchers and healthcare clinicians to promote comprehensive, accessible palliative care in the home setting.

This study has some important limitations to consider. First, the study sample was relatively small and only included perspectives from one metropolitan area. Individuals in other parts of the country might have different experiences and perspectives. Second, due to inability to provide informed consent, interviews for patients with cognitive impairment were conducted with a caregiver, thus this sub-analysis does not directly capture the perspective of patients living with dementia. Future studies should include the perspective of PLWD to allow for a better insight into the challenges they face living at home. Third, while we sampled for thematic saturation in the primary study, this is not the case for this sub-analysis. Nevertheless, many of the themes identified resonate with prior studies in this area [17]. Our results suggest an opportunity to improve home health care for patients and caregivers by integrating palliative care and additional home-based supports, particularly for adults with dementia and their caregivers.

## Conclusion

Based on multiple perspectives, there is an opportunity to improve support for PLWD and their caregivers through HHC. Overall, each group identified challenges in caring for PLWD at home and noted the increased need for supports to allow PLWD to remain at home. In this qualitative study, our formative work identified the importance of providing anticipatory guidance (e.g., safety, advance care planning) coupled with emotional and pragmatic care supports (e.g., finding resources, navigating insurance) to sustain caregivers who are struggling with the whirlwind.

### Electronic supplementary material

Below is the link to the electronic supplementary material.

Supplementary Material 1

## Data Availability

The datasets used and/or analyzed during the current study are not publicly available due to the ethical and privacy reasons around the sensitive nature of the material but are available from the senior author (CJ) on reasonable request.
