# Supplementary material for: Care challenges of home health patients living with dementia: a pathway forward with palliative care
Source: BMC Palliat Care. 2023 Aug 29;22:122. doi: 10.1186/s12904-023-01247-9 (PMC10464392; doi:10.1186/s12904-023-01247-9)
Supplement: Supplementary file 1 — Supplementary Material 1 [file 12904_2023_1247_MOESM1_ESM.pdf]

<u>PATIENTS Interview Guide</u>

Patient-level characteristics and transitional needs

For hospitalized patients:

1. Tell me what brought you into the hospital just the other week.

2. What was it like as you got ready to leave the hospital?
    a. What was important to you as you got ready to leave the hospital?
    b. What concerns did you have?
    c. What did you understand about how home health was going to help you?
    d. What kind of conversations did providers in the hospital have with you about what to expect going home? (*probe for whether talked with inpatient palliative team, what long term care plans were…*)

3. How have things been going since you have been out of the hospital?
    a. How has home health supported you?
    b. [**if no longer receiving home health** – ask remainder of interview in past tense] What made you decide to stop receiving home health care?

For outpatient patients:

4. Can you tell me about what brought you to home health?
    a. What did your providers tell you about home health?
    b. Probe for expectations, reason HH was added

5. Who have you been seeing from home health since you've been home (what kind of providers- nurses, NP, PT, OT, social worker)?
    a. What concerns have you been talking about/working on with them?
    b. What concerns has your caregiver talked about with them?
    c. What is/are the home health providers' perspective on how you're doing? What do they tell you about how you're doing?

6. Tell me about how the frequency of visits from home health feels to you. Is it happening enough, not enough, or too much?
    a. How would more frequent visits from your home health <u>nurse</u> help you?
        i. How might that help manage your symptoms?
    b. How would more frequent visits from your home health <u>social worker</u> help you?

7. How does it feel having healthcare providers come to the home? (*probe for comfort, acceptability)*
    a. How has the COVID-19 pandemic affected your thoughts about home-based care, or how comfortable you are having them come to your home?

Symptom management and provider follow up

8. What kind of symptoms are you experiencing right now that you would most like the home health nurse to focus on?

9. What kind of support would help you with medications right now? (*from nurse, etc.)*

10. What kind of support do you feel like your caregiver could use right now from home health?

11. Who is the main provider that manages your care when you are not in the hospital? *(NP, PCP, Cardiologist, Oncologist…)*
    a. Have you had any follow up with them since you've been home so far?
        i. For outpatient – ask about follow up with PCP sine starting HH
    b. Has your home health team talked with this provider at all? About what?

12. How soon would you prefer to see/connect with your primary medical provider after leaving the hospital? (Is one week too soon or not soon enough, two weeks or more, etc.?)
    a. **Omit for outpatients**

13. I understand COVID-19 has affected a lot of things, including how people are able to connect with their providers. Have you ever used telehealth video appointments with your medical providers?
    a. How did it go? What was that experience like?
    b. What is hard about connecting with your providers that way? (*wifi, etc.*)
    c. What are your thoughts about doing a telehealth visit with your home health nurse (who would be there with you in the home) and your primary medical provider (who would be connected by video call)?
        i. What would be valuable about that?
        ii. What would be hard or less desirable about that?

Advance care planning

I now have a few questions about advance care planning. Advance care planning is defined as the process of planning for future medical care based on your personal values and preferences. It can include talking with loved ones and medical providers about what you might want if you could not speak for yourself in a medical situation. It can also include choosing a power of attorney or other documents like a living will or advance directive.

14. Tell me about any experiences you've had talking about advance care planning. Have you talked about this before? With who? When? How was that?
    a. What decisions did you make?
    b. Did you complete any advance directives?
        i. Did you share your decisions with anyone? (*family, medical provider*?)
    c. How involved has your caregiver been in your advance care planning? How much do you work together on this?
    d. Have you ever talked about this/how much do you talk about this topic with a clinician or healthcare provider?

15. How do you feel about the idea of talking about advance care planning with a home health clinician like a home health social worker? Would this be something of interest to you? (*probe for comfort, acceptability*)
    a. What do you think would be valuable about talking about this with a home health social worker? Or how do you think a home health clinician could help support your advance care planning/goals of care planning?

16. If the time comes where your care needs change or your preferences about your care change, who on your care team would you feel most comfortable talking to about that?

Closing questions

17. If you were to be re-hospitalized and come back home with home health again, what would you want to be different next time as far as the support you received around that transition back home?
    a. **For outpatients – If you needed home health again in the future…..**
    b. **Probe – what would you want other home health patients who are getting a new home health service to know?**
    c. **Probe – What would you want providers to know about home health (what it is like to receive home health)?**

18. What other support or services would help you right now? Are there other resources or support you could use? (*from home health care, primary care, etc*.)
    a. What about other support or services needed for your caregiver? (respite, etc.)

19. Is there anything else that came up today that you'd like to add?
